# Potential Effects of Naringenin on Hepatic Lipid Accumulation and Lipogenesis-Related Gene Expression in High-Fat Diet-Fed Rats

**DOI:** 10.3390/ijms27219453

**Published:** 2026-10-23

**Authors:** Sarah Ahmed Alshehri, Magdi A. Osman, Ghada Alnafisah, Shaheerah Asiri, Shaista Arzoo, Laila Naif Al-Harbi

**Affiliations:** 1 Department of Food Science and Nutrition, College of Food and Agricultural Sciences, King Saud University, Riyadh 11451, Saudi Arabia; 443204280@student.ksu.edu.sa (S.A.A.); magdios@ksu.edu.sa (M.A.O.); sarzoo@ksu.edu.sa (S.A.); 2 College of Medicine, King Saud University, Riyadh 12372, Saudi Arabia; galnafisah@ksu.edu.sa (G.A.); sasiri@ksu.edu.sa (S.A.)

**Keywords:** naringenin, high-fat diet, MASLD/NAFLD, obesity, hepatic lipid metabolism, ChREBP, SREBP-1c

## Abstract

This study investigated the protective effects of naringenin (NAR) against metabolic dysfunction-associated steatotic liver disease (MASLD) in male Wistar rats fed a high-fat diet (HFD). We fed rats a high-fat diet for a total of 12 weeks, with oral NAR administered daily from weeks 9–12 (*n* = 8 rats/group). The HFD group (from week 9) were divided into four experimental groups: HFD (control); HFD+ 50 mg/kg NAR; HFD+ 100 mg/kg NAR; and HFD+ 200 mg/kg NAR. In addition, rats receiving standard diet (from week 9) were divided into two groups: a normal control (NC) group (i.e., standard diet + vehicle) and an NC+ 200 mg/kg NAR. NAR was orally administered for the last 4 weeks. We observed that the increasing doses (100 and 200 mg/kg) of NAR decreased body mass index (BMI), liver function markers, fasting plasma glucose and insulin, and HOMA-IR, and improved hepatic and serum lipid profiles. At 200 mg/kg, NAR reduced body weight by about 20.4%, BMI by 19.2%, serum ALT by 24%, AST by 43%, fasting glucose by 21%, fasting insulin by 44%, HOMA-IR by 56%, serum TG by 37%, TC by 41%, and LDL-c by 25%, and it increased HDL-c by 20%, relative to the HFD group. Similarly, it lowered the hepatic TG by 35.1%, TC by 28.4%, and LDL-C by 30.3%, while raising HDL-C by 31.8%. Histopathological examination of the liver revealed enhanced hepatocellular structure, reduced cytoplasmic vacuolation, decreased immune cell infiltration, and reduced injury scores. Furthermore, NAR was associated with improved antioxidant status, demonstrated by increasing the levels antioxidant mediators such as mRNA Nrf2, GSH, GSH-Px, SOD, and CAT in the liver. Moreover, it suppressed inflammatory mediators, including TNF-α, IL-1β, IL-6, and mRNA NF-κB, while elevating the anti-inflammatory cytokine IL-10. NAR was associated with increased hepatic SIRT1 and decreased PPARγ, ChREBP, and SREBP-1c mRNA levels. The more pronounced effect of NAR was observed at 200 mg/kg. These results of our study suggest the potential of NAR as a therapeutic candidate for MASLD, improving metabolic parameters and reducing hepatic lipid accumulation and inflammation associated with MASLD- like model, alongside improved antioxidant status and changes in SIRT1 and ChREBP/SREBP-1c expression.

## 1. Introduction

The rising prevalence of obesity, type two diabetes (T2D), and metabolic diseases has significantly heightened the risk of metabolic dysfunction-associated steatotic liver disease (MASLD), which is currently recognized as a major health issue globally [1]. In the 1980s, the disease was initially invented under the term “non-alcoholic fatty liver disease” (NAFLD) and non-alcoholic steatohepatitis (NASH) [2,3]. NAFLD was characterized by excessive hepatic fat accumulation in more than 5% of hepatocytes without other causes, such as alcohol intake, viral infections, or specific medications [4,5]. MASLD requires the presence of hepatic steatosis together with at least one of five cardiometabolic risk factors (i.e., overweight/obesity, hyperglycemia or T2D, hypertension, hypertriglyceridemia or low HDL-C) [6]. Given the close association between chronic liver conditions and metabolic syndrome (MetS), a multi-society consensus was reached to replace the terms NAFLD and NASH with MASLD and metabolic dysfunction-associated steatohepatitis (MASH) in 2023 [7]. MASLD prevalence is rising worldwide, with an estimated rate of 38% among adults. By 2040, the global prevalence of MASLD in adults is projected to exceed 55% [8]. In Saudi Arabia, the prevalence of MASLD was estimated at 43.95% among adults over 20 years in 2019 [9]. The disease may escalate from simple steatosis to MASH, leading to cirrhosis and ultimately hepatocellular cancer [10]. Pathological features of MASLD and MASH can be assessed histologically as hepatic steatosis, hepatocyte ballooning, lobular inflammation, and fibrosis [11,12].

At present, excessive caloric intake and insufficient physical activity result in obesity and IR, which are closely associated with hepatic steatosis and the progression of MASLD [13,14]. In individuals with obesity, MASLD develops through a complex interplay between adipose-tissue dysfunction and dysregulated hepatic lipid metabolism that interact to promote hepatic steatosis [15].

In the United States, resmetirom and semaglutide are FDA-approved for adults with noncirrhotic MASH and moderate to advanced fibrosis (stages F2–F3) with maintaining a healthy diet and exercise [16]. By contrast, no medication has been approved specifically for patients with isolated steatosis without MASH or significant fibrosis [17]. Nevertheless, interest is growing in bioactive compounds, including dietary polyphenols and other natural products that may influence critical pathogenic pathways (e.g., *de novo* lipogenesis (DNL), mitochondrial dysfunction, oxidative stress, and inflammation. These compounds may eventually contribute to stage-specific therapeutic approaches across the MASLD spectrum, although most remain investigational [18].

Hepatic DNL is an important contributor to intrahepatic triglyceride accumulation and therefore represents a potential therapeutic target. DNL is regulated primarily by two transcription factors: sterol regulatory element-binding protein-1c (SREBP-1c), which is mainly activated by insulin signaling, and carbohydrate-responsive element-binding protein (ChREBP), which responds primarily to glucose and other carbohydrate-derived metabolites [19,20]. These two transcription factors act synergistically to induce the expression of lipogenic genes, including acetyl-CoA carboxylase alpha (ACACA), fatty acid synthase (FASN), stearoyl-CoA desaturase 1 (SCD1), and pyruvate kinase, liver, and red blood cell (PKLR) [21]. Increased expression or activity of these lipogenic pathways has been reported in experimental diet-induced NAFLD models [22]. Post-translational modifications, including acetylation, may further regulate the stability, localization, and transcriptional activity of SREBP-1c and ChREBP [23]. However, excessive DNL may contribute to triglyceride accumulation and lipotoxic stress, which can promote oxidative stress and inflammation and facilitate progression from simple steatosis to MASH and subsequent fibrosis. However, this progression is multifactorial and is not caused by DNL alone [13,21].

Sirtuin 1 (SIRT1), a key member of the sirtuin family, plays important roles in regulating hepatic lipid metabolism, oxidative-stress responses, inflammation, and insulin sensitivity. It exerts these effects by deacetylating transcription factors and other regulatory proteins [24,25]. Reduced SIRT1 expression or activity has been associated with hepatic lipid accumulation and metabolic disturbances, whereas SIRT1 activation may improve mitochondrial function, promote autophagy, and alleviate oxidative and inflammatory stress [24,26]. Therefore, SIRT1-related pathways may represent a promising strategy for investigating the prevention and treatment of fatty liver diseases.

Citrus flavonoids, including naringenin, have attracted considerable interest as potential agents for the management of metabolic disorders [27,28]. Naringenin (NAR; 4′,5,7-trihydroxyflavonone) is a citrus-derived flavanone, which is particularly abundant in grapefruit and sour orange and exhibits diverse biological activities [29]. Previous studies have reported potential hepatoprotective, anti-inflammatory, anticancer, antidiabetic, and anti-obesity effects of NAR [29,30]. In animal studies, NAR has been shown to modulate various biological pathways associated with MASLD-like phenotype, reduce hepatic steatosis, and alleviate HFD-induced liver injury [31]. In HFD-fed animals, dietary NAR supplementation has been reported to reduce weight gain and improve serum triglyceride levels, glucose tolerance, and hyperglycemia [25,32]. Daily NAR administration has also been shown to lower fasting plasma glucose and improve lipid profiles in ApoE-/-mice [25], as well as reduce body weight, hypertriglyceridemia, inflammation, and oxidative stress in HFD-fed rats [33,34]. NAR may attenuate HFD-induced NAFLD through AMPK activation and autophagy modulation [31]. Naringin, the sugar-bound form which naringenin is derived, has shown to lower hepatic and SREBP-1c protein levels, along with downstream lipogenesis and TG-synthesis enzymes, in a fructose-fed rat model, with treatment given only in the last 4 weeks of 12-week exposure fructose exposure [35]. However, to our knowledge, the effect of naringenin on hepatic ChREBP and SREBP-1c expression in high-fat diet-fed animals has not been directly examined. Therefore, this study aimed to evaluate the effects of NAR on HFD-induced MASLD-like changes in rats, focusing on hepatic lipid accumulation and the mRNA expression of ChREBP and SREBP-1c.

## 2. Results

### 2.1. Effects of NAR Treatment on Body and Liver Weights and Food Intake

HFD-fed rats showed significantly high final body weight, and body mass index (BMI), as well as in liver weight and liver index, compared to the NC and NC+ NAR groups (*p* < 0.05; Table 1). With increasing NAR doses, we observed that BMI, liver weight, and liver index showed progressively greater numerical reductions in HFD-fed rats (*p* < 0.05; Table 1). At 200 mg/kg, we noted that NAR significantly lowered weight gain during weeks 9–12 (63.6% less than HFD), together with lower food and energy intake, but we did not observe any significant difference in these measures from HFD at 50 or 100 mg/kg.

### 2.2. Effects of NAR on Liver Enzyme Levels and Glucose Homeostasis in HFD-Fed Rats

As shown in Table 2, HFD-fed rats exhibited significantly elevated ALT, AST, and GGT levels compared to standard diet groups (*p* < 0.0001). In the levels of these enzymes between the NC and NC+ NAR (200 mg/kg) groups (all *p* > 0.05), indicating NAR alone does not alter basal liver function. The HFD+ 50 mg/kg dose group showed significant reduction only in GGT compared to HFD group. However, we noticed that the liver enzyme levels progressively decreased in HFD-fed rats treated with both 100 and 200 mg/kg NAR (*p* < 0.05), showing significant differences from HFD and the finding was more distinct at 200 mg/kg, as the difference between control and HFD+ 200 mg/kg NAR was insignificant.

Fasting plasma glucose, insulin, and HOMA IR levels showed no significant differences between NC and NC+ NAR (200 mg/kg) groups (all *p* > 0.05). HFD feeding significantly increased these parameters compared to NC and NC+ NAR (200 mg/kg) groups (*p* < 0.05). Although, we did not observe any significance difference in these parameters between HFD and 50 mg/kg NAR + HFD groups (*p* > 0.05), but we found that, NAR at both 100 and 200 mg/kg significantly reduced fasting insulin, glucose and HOMA-IR levels versus HFD group (*p* < 0.05). The finding was more prominent at 200 mg/kg and the difference between control and HFD+ 200 mg/kg NAR was insignificant. These findings demonstrate that highest dose showed the most pronounced hepato-protective effects and improvement of insulin resistance indices in HFD induced MASLD-like model.

### 2.3. Effects of NAR on Serum and Liver Lipid Parameters

Data shown in Table 3 demonstrate that serum and hepatic lipid profile levels showed no significant differences between the NC and the NC+ NAR groups. Compared to the NC group, HFD-fed rats exhibited significantly elevated serum TC, TG (both *p* < 0.0001), and LDL-c levels (*p* < 0.01), along with reduced HDL c (*p* < 0.05). NAR treatment at 100 and 200 mg/kg significantly reduced serum TC, TG, and LDL-c (all *p* < 0.05) and significantly increased HDL-c, whereas the 50 mg/kg dose showed no significant effect compared to the HFD group (*p* > 0.05). Similarly, hepatic lipid analysis showed a significant increase in TG, TC, and LDL-c levels and a decrease in HDL-c in HFD-fed rats compared with the NC and NC+ NAR groups (all *p* < 0.05). NAR at 100 and 200 mg/kg significantly ameliorated these parameters (*p* < 0.05), while the 50 mg/kg dose had no significant effect as compared to the HFD group (*p* > 0.05). We observed that among the NAR-treated groups, higher doses generally exerted more pronounced effects, with the 200 mg/kg dose showing the most pronounced improvement in serum and hepatic TG, TC, and LDL-c, and the greatest increase in HDL-c, compared with the HFD group, showing insignificant difference when compared with control.

### 2.4. Effects of NAR on Selected Markers of Hepatic Oxidative Stress

**Figure 1:**
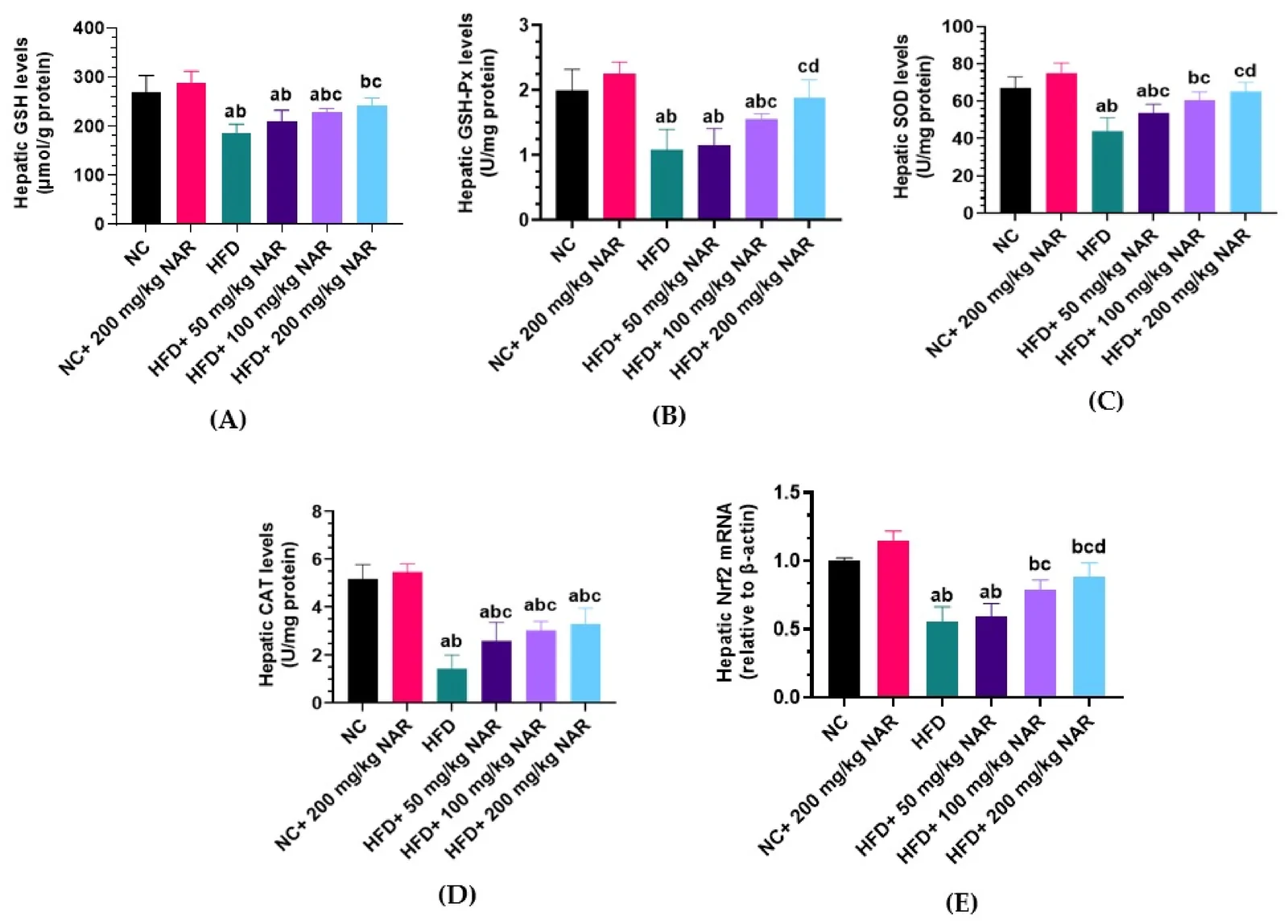
Changes in the activity of oxidative stress-related biomarkers in liver homogenate of all groups of rats. (**A**) Reduced glutathione (GSH), (**B**) glutathione peroxidase (GSH-Px), (**C**) superoxide dismutase (SOD), (**D**) catalase (CAT), and mRNA levels of (**E**) nuclear factor erythroid 2-related factor 2 (Nrf2). Data are reported as mean ± SD and significance levels were presented as *p* < 0.05. Statistical comparisons are denoted by letters as follows: (a) compared to control group, (b) compared to control group treated with NAR (200 mg/kg), (c) compared to HFD group, (d) compared to HFD rats treated with NAR (50 mg/kg). A letter on a bar means that group differs significantly (*p* < 0.05) from the group listed for that letter. Abbreviations: NC: normal control; HFD: high-fat diet; NAR: naringenin.

In the HFD group, rats showed significantly decreased hepatic antioxidant activities of GSH, GSH-Px, SOD, and CAT (all *p* < 0.0001). Nrf2 expression was significantly reduced (*p* < 0.001) compared with NC group (Figure 1A–E). NAR treatment at 200 mg/kg dose improved these antioxidant parameters approaching those of the NC group. GSH levels and GSH-Px activity were significantly higher compared with the HFD for GSH (*p* < 0.05 and *p* < 0.01, respectively) and GSH-Px (*p* < 0.05 and *p* < 0.0001, respectively) at 100 and 200 mg/kg (Figure 1A,B). NAR also significantly increased the activities of SOD and CAT at all doses (*p* < 0.05, *p* < 0.001, and *p* < 0.0001), respectively (Figure 1C,D). Moreover, the higher doses of NAR (100 and 200 mg/kg) significantly increased Nrf2 expression (*p* < 0.05 and *p* < 0.01, respectively) compared with the untreated HFD group (Figure 1E). Furthermore, the levels of all measured antioxidant and Nrf2 mRNA in the NC+ NAR (200 mg/kg) group showed no significant difference compared to NC group (*p* > 0.05; Figure 1A–E). Collectively, these findings elucidate that NAR exerts a beneficial effect on metabolic regulation and bolsters antioxidant defenses during HFD exposure, supporting its hepatoprotective and anti-lipogenic potential.

### 2.5. Effects of NAR on Hepatic Inflammatory and Inflammation-Related Markers

**Figure 2:**
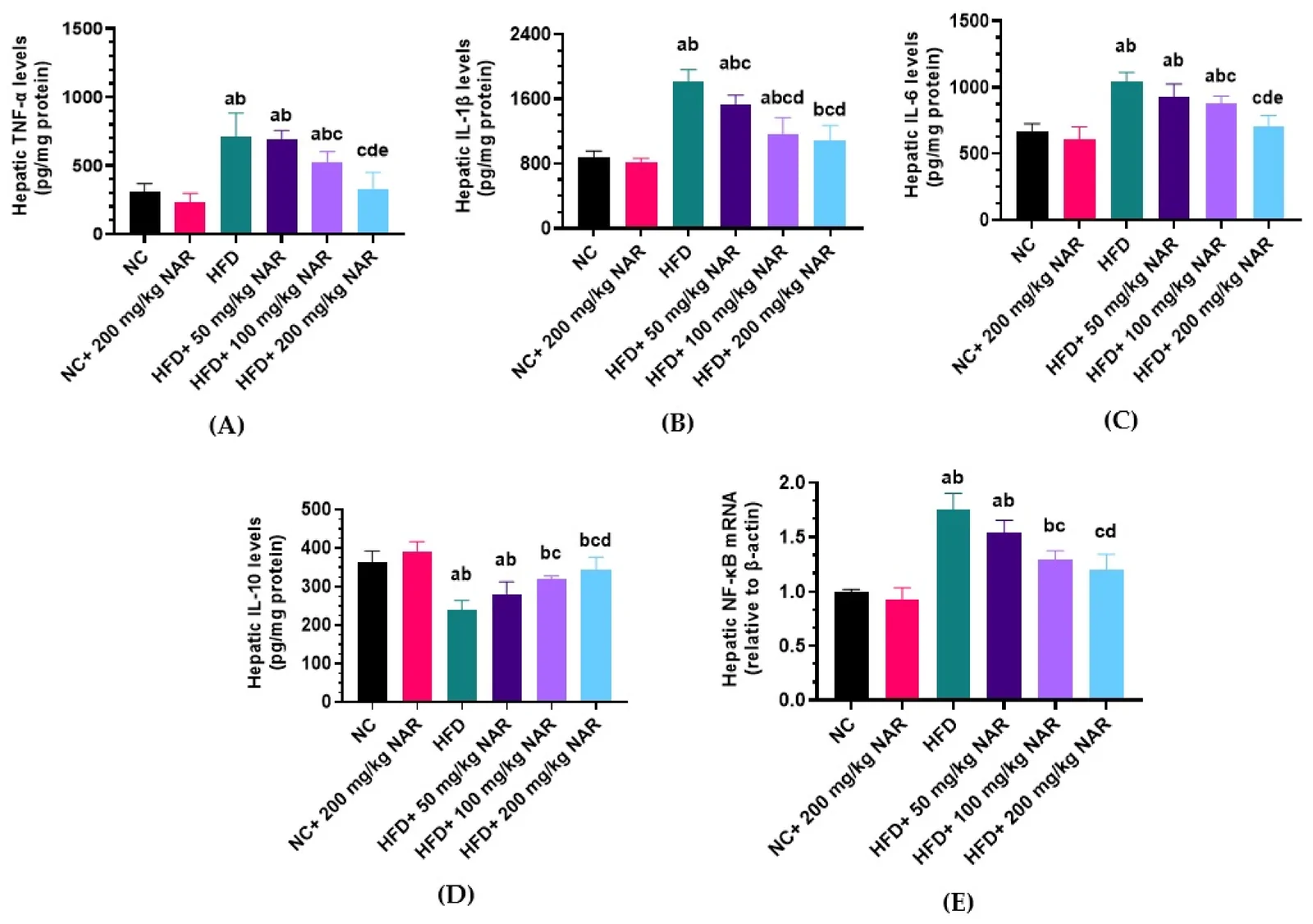
Changes in the levels of hepatic inflammatory biomarkers of all groups of rats. (**A**) Tumor necrosis factor-alpha (TNF-α), (**B**) interleukin-1beta (IL-1β), (**C**) interleukin-6 (IL-6), (**D**) interleukin-10 (IL-10), and mRNA levels of (**E**) nuclear factor-kappa B (NF-κB). Data are reported as mean ± SD and significance levels were presented as *p* < 0.05. Statistical comparisons are denoted by letters as follows: (a) compared to control group, (b) compared to the control group treated with NAR (200 mg/kg), (c) compared to the HFD group, (d) compared to HFD rats treated with NAR (50 mg/kg), and (e) compared to HFD rats treated with NAR (100 mg/kg). A letter on a bar means that group differs significantly (*p* < 0.05) from the group listed for that letter. Abbreviations: NC: normal control; HFD: high-fat diet; NAR: naringenin.

In the context of inflammation as shown in Figure 2A–E, HFD-fed rats exhibited significantly elevated hepatic levels of the pro-inflammatory cytokines IL-6, TNF-α, and IL-1β, with a marked reduction in IL-10 (all *p* < 0.0001) as compared with NC rats. We observed that, when compared with HFD, the HFD rats administered 100 and 200 mg/kg NAR significantly decreased IL-6 and TNF-α, whereas the 50 mg/kg doses had no significant impact (*p* > 0.05). Notably, hepatic IL-1β levels were significantly reduced at all administered doses of NAR (*p* < 0.05) compared to the HFD group. Furthermore, cytokine IL-10 was not significantly increased by NAR at 50 mg/kg, while treatment at 100 and 200 mg/kg significantly increased IL-10 levels (*p* < 0.001) and (*p* < 0.0001), respectively, compared with HFD group (Figure 2 A–D). These changes were accompanied by a significant increase in hepatic NF-κB (*Rela*) expression (Figure 2E) in the HFD group (*p* < 0.0001), consistent with enhanced inflammatory signaling. However, NAR treatment (100 and 200 mg/kg) significantly reduced NF-κB (*Rela*) mRNA expression compared with the untreated HFD group. Finally, the levels of all measured cytokines and NF-κB mRNA in the NC+ NAR (200 mg/kg) group showed no significant difference compared to NC group (*p* > 0.05; Figure 2A–E).

### 2.6. Effects of NAR on Hepatic Central Regulators of Lipid Metabolism

**Figure 3:**
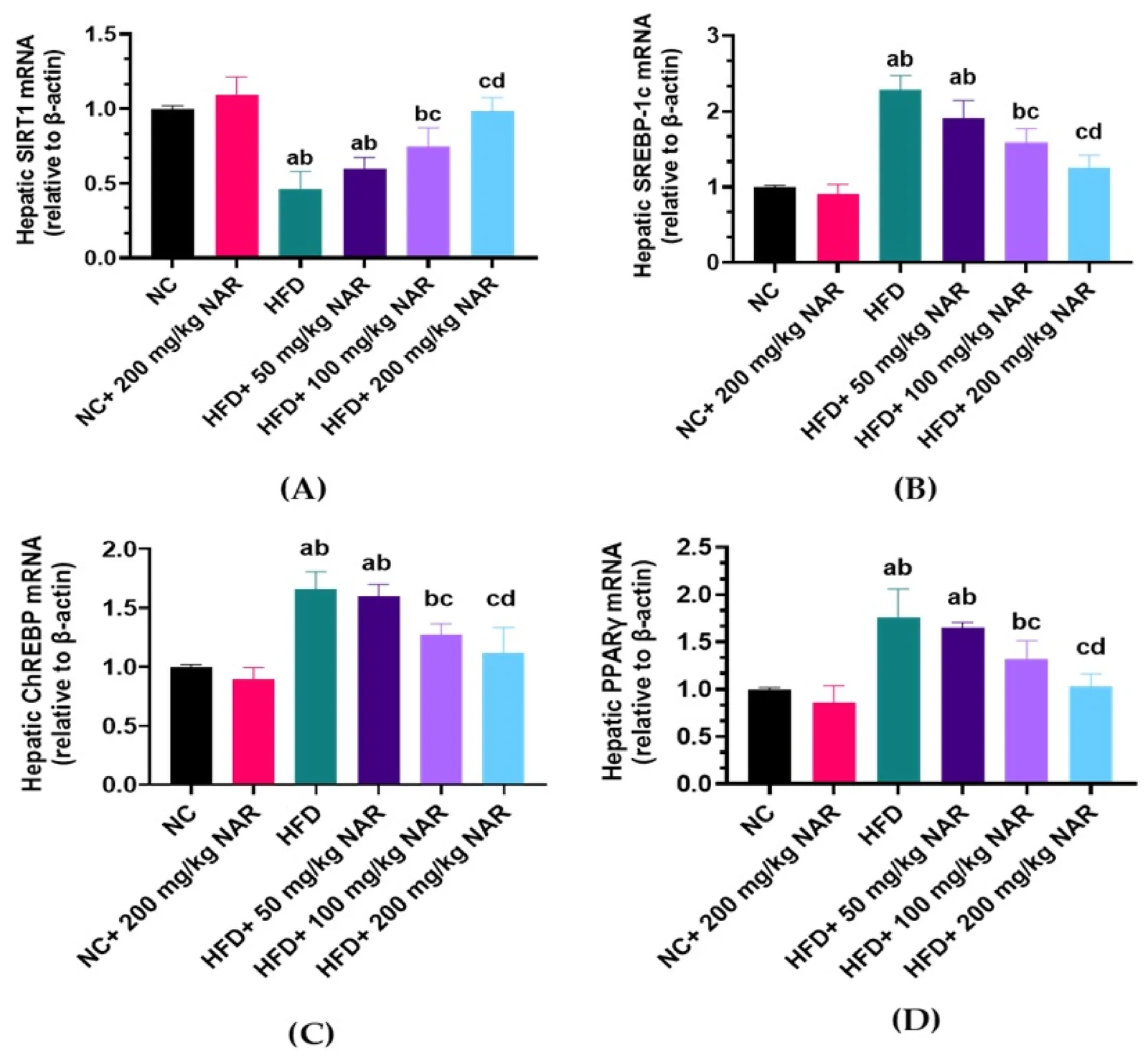
Effects of NAR treatment on hepatic expression of metabolic and lipogenic regulators. Hepatic mRNA levels of (**A**) SIRT1, (**B**) SREBP-1c, (**C**) ChREBP, and (**D**) PPARγ were determined by quantitative real-time PCR. Relative expression was calculated using the 2−ΔΔCt method with β-actin as the endogenous reference gene and the designated control group as the calibrator. Each RNA/cDNA sample was measured in technical triplicate, and the technical replicates were averaged before animal-level ΔCt values were used for the statistical analysis. Data are presented as mean ± SD. A letter on a bar means that group differs significantly (*p* < 0.05) from the group listed for that letter. Abbreviations: NC: normal control; HFD: high-fat diet; NAR: naringenin.

There were no significant differences in the expression of PPAR-γ, SREBP-1c, ChREBP, and SIRT1 (*p* > 0.05) between the NC and NC+ 200 mg/kg NAR groups (Figure 3A–D). We found that quantitative RT-PCR analysis revealed alterations in the expression of genes involved in hepatic lipid metabolism and metabolic regulation following HFD feeding. HFD feeding markedly increased hepatic SREBP-1c (*p* < 0.0001), ChREBP (*p* < 0.001) and PPAR-γ (*p* < 0.001), and decreased SIRT1 expression compared with the normal control group. We observed that treatment with NAR at 100 and 200 mg/kg significantly attenuated the HFD-induced alterations compared with HFD group, whereas 50 mg/kg did not produce a significant change. Notably, the expression level of SIRT1, PPAR-γ, SREBP-1c, and ChREBP observed in 100 and 200 mg/kg did not differ significantly from those of the NC group and among the NAR treated groups, the 200 mg/kg dose showed the most pronounced numerical improvement in the expression of these genes.

### 2.7. Effects of NAR on Histological Characteristics of Hepatocytes in Rats

**Figure 4:**
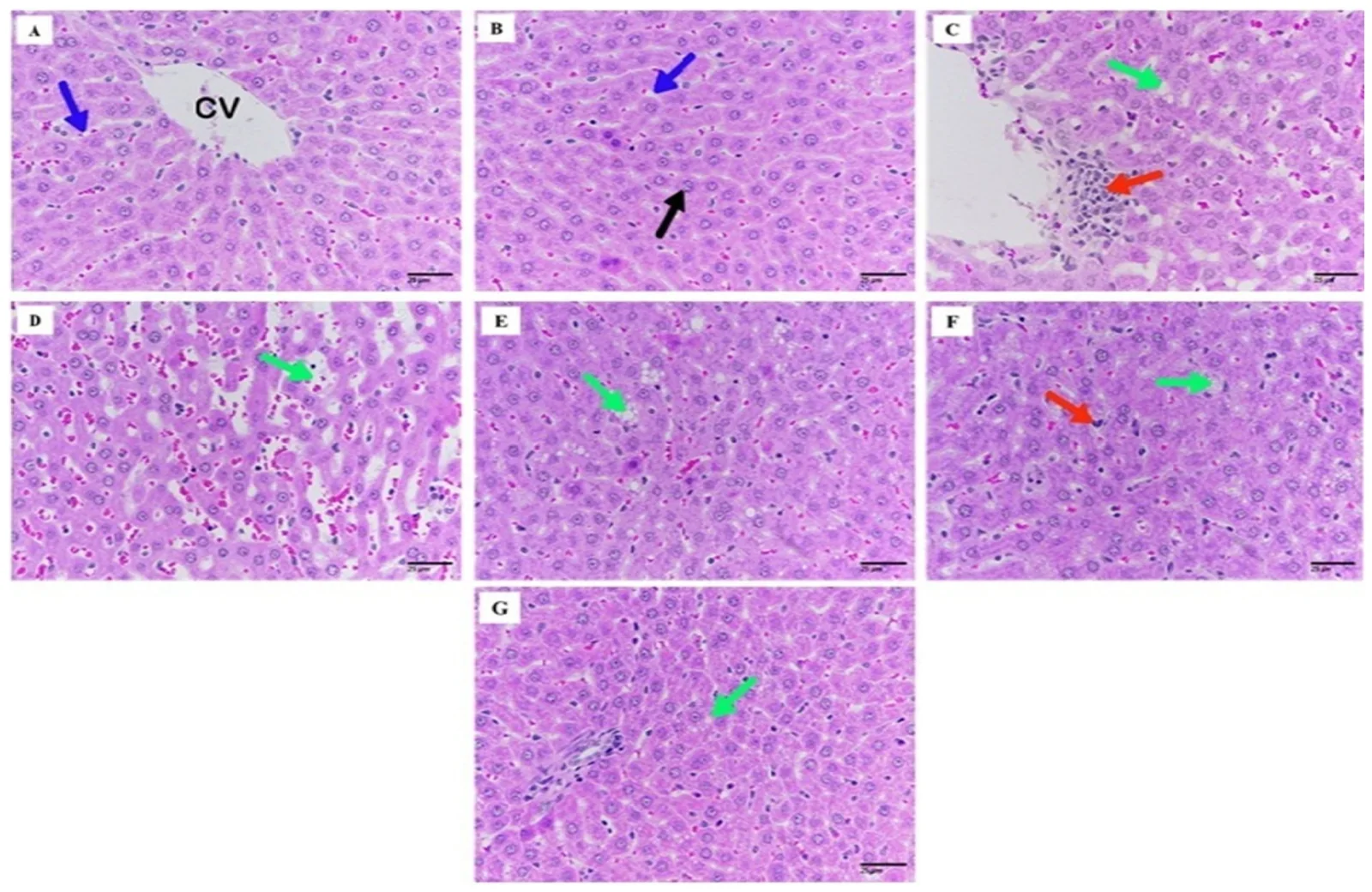
Photomicrographs of H&E-stained liver sections from rats in all experimental groups (scale bar = 25 μm; magnification × 400). Groups are defined as follows: (**A**) Normal control rats fed a standard diet (SD) showing normal hepatocyte architecture, central vein (CV), and blood sinusoids (blue arrow). (**B**) Liver section from the NC+ NAR 200 mg/kg group showing no apparent pathological changes in the hepatocytes (black arrow) or sinusoids (blue arrow). (**C**,**D**) Liver sections from HFD-fed rats showing increased inflammatory-cell infiltration around the portal tracts (portal inflammation; red arrow), numerous cytoplasmic vacuoles of varying sizes and marked dilation of the blood sinusoids (green arrow). (**E**) Liver section from the HFD+ NAR 50 mg/kg group showing reduced cytoplasmic vacuolation, with mild sinusoidal dilation in some areas. (**F**,**G**) Liver sections from HFD-fed rats treated with NAR at 100 and 200 mg/kg, respectively, showing substantial restoration of hepatocellular architecture and reduced inflammatory cell infiltration.

Histological evaluation of liver sections is shown in Figure 4A–G across all experimental groups. The liver tissues collected from the control (NC) and NC+NAR (200 mg/kg)-treated rats displayed a normal hepatic architecture, including intact hepatocytes, central veins, sinusoids, and centrally located nuclei, with no signs of morphological abnormalities (Figure 4A,B). In contrast, the livers of HFD-fed rats exhibited prominent macrovesicular steatotic features, marked dilatation of hepatic sinusoids, and focal inflammatory cell infiltration, together with prominent cytoplasmic vacuolation (Figure 4 C,D). These findings are also supported by histopathological assessment.

Meanwhile, the livers of HFD+ NAR-treated rats (50 mg/kg) showed improvement in hepatic morphology, reduced cytoplasmic vacuolation, normally appearing hepatocytes, and sinusoids were seen in the livers (Figure 4E). Further morphological improvement was observed in HFD-fed rats administered NAR at 100 and 200 mg/kg, characterized by more preserved sinusoidal morphology, reduced vacuolar changes, and decreased inflammatory cell infiltration (Figure 4F,G).

### 2.8. Effect of NAR on Histopathological Score System Damage

Histopathological examination revealed no detectable hepatic alterations in the control and NAR groups (Table 4). In contrast, HFD sections showed significant increases in all pathological score criteria with a total histopathological score of 14.6. The HFD+NAR 50 mg/kg group displayed some reduction in edema, accompanied by a significant decline of total histopathological score 12. Treatment with NAR at 100 mg/kg produced significant decline of cytoplasmic degeneration and hemorrhage, with a marked decrease of total score of 10.5. The HFD+NAR 200 mg/kg group exhibited the most significant reductions in microvesicular steatosis, cytoplasmic degeneration, inflammatory cell infiltration, hemorrhage, and edema, with total decline in histopathological score of 9.2.

## 3. Discussion

MASLD, previously known as NAFLD [36], is recognized as one of the most widespread metabolic disorders globally, closely linked to obesity, insulin resistance, dyslipidemia, and metabolic syndrome. The pathogenesis of MASLD is intricate, involving the dysregulation of hepatic lipid metabolism, oxidative stress, mitochondrial dysfunction, and chronic inflammation [37]. Natural compounds, particularly polyphenols such as NAR, have emerged as a potential therapeutic agent. NAR possesses diverse biological activities and effective therapeutic agents for the management of MASLD [29,30]. Several studies have reported that NAR exhibits positive health benefits associated with hepatoprotective, anti-inflammatory, antidiabetic, and anti-obesity effects [38,39,40,41]. Among the primary molecular contributors to hepatic steatosis are transcription factors that govern *de novo* lipogensis (DNL). In the present study, we examined the effects of three doses of NAR (50, 100, and 200 mg/kg) in high-fat diet-fed rats with MASLD-like features, focusing on hepatic lipid accumulation, oxidative stress, inflammation, and the expression of lipogenesis-related genes.

NAR exists in the form of an aglycone (basic flavanone structure without sugars) and has better oral bioavailability than its glycoside form, naringin [42]. Its antioxidant have been attributed to the 4′, 5′, and 7′ positions of the three key hydroxyl groups and these structural characteristics may contribute to the biological activity of NAR [42]. HFD regimens are widely used (45–75% energy from fat), and they are extensively employed in MASLD prevention and treatment studies due to their strong association with obesity and metabolic dysfunction [43]. Excessive HFD intake in both humans and rodents is a major contributor to MASLD onset and progression through the promotion of hepatic lipid accumulation and persistent activation of hepatic DNL [44,45]. HFD-based rodent models are extensively used because they can reproduce many of the metabolic and histological features of human MASLD [46,47].

In our study, we established an HFD rat model of obesity-associated MASLD-like disease for 12 consecutive weeks, followed by NAR treatment during the last 4 weeks at three doses (50, 100, and 200 mg/kg). The validity of this model is supported by previous work showing that chronic HFD feeding for 12 weeks significantly increased body weight, liver weight, lipid accumulation, fasting glucose, insulin levels, and HOMA-IR in rats [48,49,50,51]. Consistent with earlier reports, our findings showed that HFD feeding increased TG and TC while decreasing HDL-C in HFD-fed rats [34,52]. In addition, the histological analysis revealed marked intracytoplasmic vacuolization and inflammatory cell infiltration, accompanied by elevated serum transaminases, indicating the development of the characteristic features of MASLD after 12 weeks of HFD exposure.

Although we did not observe marked fatty infiltration in the representative H&E sections, the histopathological scoring confirmed the presence of both macrovesicular and microvesicular steatosis in the HFD group (Table 4). The severity of hepatic steatosis in diet-induced rodent models varies according to the animal strain, dietary composition, fat source, feeding duration, and the inclusion of fructose or cholesterol. HFD-only regimens may produce less pronounced histological changes than Western diet or high-fat/high-fructose models [53,54,55]. In our present study, the observed histopathological alterations such as steatosis, cytoplasmic degeneration, infiltrative changes, hemorrhage, edema, together with the biochemical changes in liver transaminases and lipid profile, supported the occurrence of HFD-associated hepatic injury.

Hepatic fat accumulation results from an imbalance in lipid acquisition and disposal, including increased fatty acid uptake, reduced export, impaired β-oxidation, and enhanced DNL [13,56]. In our study, in the HFD-induced rat model, NAR at 200 mg/kg reduced food and energy intake, the lower weight gain cannot be attributed to a direct metabolic effect of NAR alone; a pair-fed control group would be needed to separate these effects, while NAR at 100 and 200 mg/kg doses significantly reduced BMI, and all doses of NAR showed reduced liver weight and liver index as the difference was significant with the HFD group. At the metabolic level, treatment with NAR at 100 and 200 mg/kg significantly reduced fasting blood glucose, hyperinsulinemia, and IR, while improving the serum lipid profile, by decreasing total cholesterol, triglycerides, and LDL and increasing HDL. The more pronounced effect was observed at treatment with 200 mg/kg. These findings are supported by previous research by Cai et al. [52], which demonstrated that NAR reduces body weight, liver weight, and IR in HFD-fed rats but without affecting food intake. Previously, oral administration of NAR at 100 mg/kg for 4 weeks produced comparable anti-obesity effects in animal models, particularly by reducing body weight gain [57]. Liu et al. [34] also reported that oral NAR administration at doses of 50 and 100 mg/kg for 4 consecutive weeks reduced body weight gain and improved hypertension in HFD-fed rats compared with control animals. However, in our study, the 50 mg/kg dose did not significantly affect body weight, possibly because of differences in HFD composition between the two studies. In addition, previously, *Ldlr-/-* mice fed a high-fat, cholesterol diet supplemented with 3.0% NAR for 12 weeks showed a reduction in body weight, plasma improvement in lipids, and IR [33]. Moreover, Namkhah et al. [58] reported that NAR (200 mg/kg) treatment improved lipids and anthropometric parameters in NAFLD patients, including TC, TG, LDL-C, HDL-C, and body weight. Moreover, the treatment with NAR (100 and 200 mg/kg) decreased the serum liver enzymes, oxidative stress, and hepatic cytokines.

Our results showed that NAR treatment at 50 mg/kg had little positive effect on alleviating MASLD-like liver injury. Histologically, increasing doses of NAR therapy diminished the occurrence of cytoplasmic vacuoles, portal inflammation, and dilation of blood sinusoids and infiltration of inflammatory cells. These results are in line with previous studies that have shown that NAR decreases serum levels of triglycerides and total cholesterol and improves liver function [31]. Serum GGT has been used as an additional biochemical indicator in rat models of MASLD induced by an HFD [59]. NAR reduced elevated GGT concentrations in Taxol-induced hepatic injury, although this was not a model of NAFLD or HFD [60].

One of the main findings of our study is that NAR at 100 and 200 mg/kg was associated with lower hepatic expression of ChREBP and SREBP-1c in HFD-fed rats. We observed that the hepatic mRNA expression of SREBP-1c, ChREBP, and PPARγ, which are key transcription factors regulating lipogenesis, was significantly higher in the HFD group than in the control group. The major activator of SREBP-1c is insulin signaling, which induces the expression of fatty acid synthesis-related genes such as acetyl-CoA carboxylase (ACC) and fatty acid synthase (FAS) [51]. Similarly, ChREBP and PPARγ are important nutrient-sensitive transcription factors that coordinate to regulate glucose and lipid metabolism, particularly in response to increased carbohydrate intake [59]. These transcription factors are excessively activated and are strongly associated with liver lipid accumulation and progression of NAFLD [59,60].

The expression of PPAR-γ, SREBP-1c, and ChREBP in the liver was significantly decreased by the NAR treatment (100 and 200 mg/kg) compared with the untreated HFD group. This decreased expression indicates that NAR may inhibit hepatic lipogenesis at the transcriptional level, leading to decreased lipid accumulation in hepatocytes. More recently, it has been demonstrated that flavonoids could inhibit the expression of lipogenic genes and improve lipid metabolism through affecting key transcriptional regulators [61,62]. NAR may help attenuate hepatic steatosis by reducing the expression of these transcription factors, potentially contributing to reduced fatty acid and triglyceride synthesis.

NAR also had a significant effect on the hepatic SIRT1expression. It is a NAD+ -dependent deacetylase that plays a significant role in the regulation of cellular energy homeostasis. Previously, it has been reported that SIRT1 is involved in several metabolic processes, including metabolic functions including mitochondrial biogenesis, fatty acid oxidation and glucose metabolism [63]. The accumulating evidence suggests that SIRT1 deacetylates key transcriptional regulators to regulate hepatic lipid metabolism, oxidative stress and inflammation, and may therefore contribute to the prevention of the development of fatty liver disease [24,25,64,65]. In NAFLD, SIRT1 significantly impacts DNL pathways through deacetylation, controlling the function of SREBP-1c and ChREBP, suppressing lipogenesis, and promoting hepatic lipid homeostasis [24,66]. Moreover, SIRT1 has been associated with antioxidant responses through the Nrf2 pathway and to the regulation of inflammatory signaling such as NF-κB [67]. SIRT1 mediated deacetylation of downstream targets such as PGC-1α has been associated with mitochondrial function and fatty acid oxidation [68]. We found that previous studies have reported that decreased hepatic SIRT1 or systemic SIRT1 activity can aggravate HFD-induced liver fat accumulation, while SIRT1 overexpression may reduce liver damage and improve metabolic parameters under HFD feeding conditions [26,69].

It is now widely recognized that oxidative stress is an essential factor in the progression from hepatic steatosis to more severe liver injury [70]. An excess accumulation of lipids in hepatocytes increases mitochondrial β-oxidation and production of reactive oxygen species (ROS), resulting in oxidative damage to cellular macromolecules [71]. A major regulator of cellular antioxidant defense systems is the transcription factor Nrf2 [72]. Nrf2 activation is associated with its translocation to the nucleus and subsequent transcription of antioxidant genes, such as SOD, CAT, and HO-1 [72]. The present study demonstrated that Nrf2 expression was significantly reduced in the HFD group, suggesting that antioxidant defense may got weakened, whereas NAR treatment significantly increased the expression of Nrf2 compared to the untreated HFD group, but we did not directly assess Nrf2 nuclear translocation and downstream pathway activation, so the present findings do not establish Nrf2 activation as a causal mechanism.

Chronic inflammation is a major driver of hepatic metabolic disorder [73], and inflammatory responses are primarily regulated by the transcription factor NF-κB, which controls the expression of multiple pro-inflammatory cytokines, including TNF-α, IL-1β, and IL-6 [74]. In the current study, NF-κB expression was significantly elevated in the HFD group, suggesting an association with inflammatory signaling pathways in liver tissue. These findings are consistent with previous studies which reported association between high-fat diets and hepatic inflammation involving NF-κB-mediated signaling cascades [75,76].

Notably, NAR treatment led to a significant reduction in NF-κB expression, supporting an anti-inflammatory potential. Similar features have been reported for several dietary flavonoids that inhibit NF-κB signaling and decrease cytokine production in models of metabolic disorders [77]. The anti-inflammatory effect of NAR on HFD-induced liver injury is mainly achieved by regulating the balance of pro-inflammatory and anti-inflammatory cytokines, which is consistent with our results. Rats fed HFD were in a state of low-grade chronic inflammation with significant increase in hepatic concentrations of TNF-α, IL-6, and IL-1β. These results are consistent with previous studies showing that long-term HFD intake promotes inflammatory cell infiltration and the secretion of inflammatory mediators in the liver [31,78].

The reduction in NF-ĸB mRNA expression by NAR treatment is a vital finding, as NF-κB is a central regulator of inflammatory gene expression. During inflammation, NF-κB translocates to the nucleus, activating the transcription of genes associated with inflammatory response. SIRT1 deacetylates NF-κB, thereby inhibiting its transcriptional activity and reducing macrophage infiltration and the secretion of pro-inflammatory cytokines [79]. The reduction which we observed in NF-κB expression following NAR treatment (100 &200 mg/kg) may contribute to the lower TNF-α and IL-6 levels, which aligns with earlier reports highlighting NAR may modulate NF-κB signaling through IκB kinase (IKK) complex or nuclear translocation of NF-κB subunit [13,80]. But is important to note that we did not directly assess these mechanisms in our study and may contribute to anti-inflammatory effects.

Interestingly, we observed that while TNF-α and IL-6 were significantly reduced at 100 and 200 mg/kg NAR, IL-1β were significantly reduced at all doses. Our findings indicate that IL-1β responded to NAR treatment even at lower dose than TNF-α and IL-6, but it should not be interpreted as evidence of greater sensitivity to NAR. The mechanism responsible for the differences in responses of these inflammatory mediators remain unclear and so it requires further investigation. Moreover, the marked increase in IL-10 levels following NAR administration suggests that NAR may exert anti-inflammatory effects in addition to reducing pro inflammatory cytokines, which is crucial for limiting tissue damage and supporting hepatic recovery.

Recent investigations have highlighted a strong functional relationship between the Nrf2 and NF-κB pathways. Activation of Nrf2 not only enhances antioxidant defenses but also restrains inflammatory responses by inhibiting NF-κB signaling. Studies with flavonoids have shown that the modulation of the Nrf2/NF-κB axis can simultaneously alleviate oxidative stress, inflammation, and lipid accumulation in models of NAFLD [68,81]. In our present study, treatment with NAR at 100 and 200 mg/kg was associated with increased Nrf2 expression and decreased NF-κB expression together with improvements in antioxidant and anti-inflammatory markers. It suggests that changes in these expressions may contribute to hepato-protective effect of NAR but we did not directly assess their interactions and their casual roles.

It is important to note that, in our study, we tested three doses of naringenin (50, 100, and 200 mg/kg), which are far higher than what is achievable through normal grapefruit consumption. In the fruit, naringenin exists mostly as its bound form, “naringin”, while the free aglycone is present in the juice only at low or undetectable levels [82]. Once we convert our rat doses to human terms using the body-surface-area of Reagan-Shaw et al., [83] with “km = 6, corresponding to 150 g reference rat weight,” consistent with the body weight range (150 ± 20 g) of animals, used in this study, our doses gives a range correspond to roughly 560–2260 mg per day for an average adult. This range is simply impossible to reach by diet alone, since meaningful plasma naringenin levels are only observed when the compound is purified [84]. In addition to this, naringenin has an inherently poor oral bioavailability, which has motivated the development of nanodelivery formulations aimed at improving its absorption [85], and human absorption of naringenin from citrus foods is also known to vary considerably between individuals, likely due to differences in gut microbiota [86]. In line with this, the clinical trials administering naringenin have done so using purified or concentrated extract preparations rather than through ordinary dietary intake [58,87] suggesting that the hepatoprotective effects reported here may reflect the effects of naringenin as a supplement or pharmacological agent, rather than an effect readily achieved by habitual grapefruit consumption.

### Study Limitations

Although this study showed positive effects of NAR on HFD-induced MASLD-like changes, some limitations should be taken into consideration. The study was conducted in an animal model and cannot be fully extrapolated to human metabolism or the heterogeneity of human disease. We did not confirm the metabolic disturbance and hepatic steatosis in individual animals before NAR treatment. The study was limited by the 4-week treatment period, lack of long-term follow-up, and absence of pharmacokinetic or bioavailability assessments. Further studies are needed to confirm the long-term effects and pharmacological properties of NAR. We focused on a limited set of molecular analysis and did not directly investigate the upstream mechanisms of naringenin and so, more pathways are expected to contribute, and future work should address them. A further limitation in our present study is in the dietary model. Most of the difference between the HFD (D12492) and the control diet (D12450J) used in our study comes from the fat content (60% vs. 10%, mainly from lard), while sucrose (68 g) and cellulose (50 g) remain similar between the control and high-fat diet groups. Although this fat-predominant model used in our study was sufficient to induce hepatocellular vacuolation and portal inflammatory cell infiltration, it does not fully reflect the combined high-fat, high-sugar, low-fiber pattern that typically drives MASLD in humans, and the fiber source used, purified cellulose, is non-fermentable unlike the fiber found in whole foods [88,89]. So, in future, in order to better mimic human dietary patterns, it would be beneficial to incorporate added fructose and more fermentable fiber source along with elevated fat to improve translational relevance. Additionally, we did not directly measure *de novo* lipogenesis and TG esterification flux, and no causal interventions (such as pathway inhibition or gene knockdown) were performed, so the findings indicate associations rather than direct mechanisms. Apart from this, some of the metabolic and hepatic improvements observed in our present study at 200 mg/kg may have been partly secondary to reduced body weight (as there was significant reduction in weight at this dosage) rather than to direct hepatic effects, and this could not be separated in the present design. We collected blood under ketamine/xylazine anesthesia, which can influence glucose and insulin concentrations; so, the absolute values should be read with caution, although we followed the protocol for all animals. It is also important to note that HOMA-IR is an indirect surrogate estimate of insulin resistance in rats and is not equivalent to functional measurements such as the hyperinsulinemic-euglycemic clamp. Biochemical analyses used six animals per group, which limits statistical power. Also, no correction was made for multiple comparisons across endpoints, so the results should be viewed as exploratory and confirmed in larger studies. Because H&E staining was used for histopathological assessment, the observed cytoplasmic vacuolation cannot definitively establish lipid accumulation in every instance. Limited resources prevented additional validation assays on the protein level (e.g., Western blotting) and histology staining methods specific for lipids (e.g., Oil Red O). Further studies using these approaches, combined with human trials, are necessary to improve reproducibility and translational applicability of NAR-based therapies for metabolic liver diseases.

## 4. Materials and Methods

### 4.1. Drugs and Chemicals

Naringenin (NAR; C_15_H_12_O_5_, molecular weight = 272.25 g/mol), a flavonoid derived from grapefruit (*Citrus paradisi* Macf.) was purchased from Sigma Aldrich Company, St. Louis, MO, USA (natural grade, purity 98.0%, Cat. No. W530098). Dimethyl sulfoxide (DMSO; ≥99.9%) was obtained from VWR International S.A.S., Fontenay-sous-Bois, France. Tween 80 was purchased from Alpha Chemika Company, Mumbai, India.

### 4.2. Dosing and Preparation of Naringenin

Fifty mg/kg NAR dosage was selected based on previous investigations that identified it as the effective minimum therapeutic dose for hepatoprotection and NAFLD models [38,90]. The doses of 100 and 200 mg/kg/day were selected in accordance with previous studies [25,31,34]. NAR doses were freshly prepared each day in a vehicle consisting of 90% physiological saline (PS) (NaCl 0.9%), 5% DMSO, and 5% Tween 80. Formulations were prepared at concentrations of 10, 20, and 40 mg/mL to deliver NAR doses of 50, 100, and 200 mg/kg/day, respectively, at a fixed administration volume of 0.5 mL/100 g body weight. NAR was administered as a suspension and not as a clear solution. To ensure dose uniformity, the suspension was vortex-mixed immediately before each individual dose was withdrawn, which redispersed suspended particles, minimized settling and maintained a uniform dose across animals [91].

### 4.3. Ethical Statement

The Ethical Guidelines of the Research Ethics Committee at KSU, Riyadh, Saudi Arabia, approved all the animal care procedures used in this study (Ethics Reference No: SE-25–17).

### 4.4. Experimental Animals

The Experimental Animal Care Centre at the College of Pharmacy, King Saud University (KSU), Riyadh, Saudi Arabia, supplied 48 adult male Wistar rats (150 ± 20 g), aged 6–7 weeks old. Rats were housed in separate plastic cages in a room with controlled conditions (12 h of light/dark cycles, 50 ± 5% relative humidity, and 22 ± 2 °C) with free access to water and a standard pellet diet.

### 4.5. Diets and Induction of MASLD

Based on the evidence [88], that HFD providing 45–60% of total energy from fat induce clinically significant metabolic alterations in rodents, we used a standard control diet (Cat. No. D12450J; 10% kcal from fat, 3.85 kcal/g) and a high-fat diet (HFD; Cat. No. D12492; 60% kcal from fat, 5.24 kcal/g) in the study. The two commercial diets used in our study shared several ingredients, including casein, sucrose, soybean oil, cellulose, and vitamin and mineral components. However, the amounts of these ingredients differed between the two diets, resulting in different macronutrient and energy compositions. In precise, the HFD contained a higher amount of lard and no corn starch compared with the SD. The detailed composition of the two diets is provided in Table 5. Both diets were obtained from Research Diets Inc., New Brunswick, NJ, USA [92,93].

### 4.6. Experiment and Intervention Protocol

Following 1-week acclimatization on AIN-93G purified diet [94], rats were randomly allocated into two dietary groups: normal control (NC) group (*n* = 16) receiving D12450J (10% kcal fat), and HFD group (*n* = 32) receiving D12492 (60% kcal fat) for 12 weeks. At the end of week 8 (Table 6), rats were ranked by body weight within each diet and allocated to the experimental groups by body-weight matching. We divided the HFD-fed rats (*n* = 32) into eight strata of four rats of similar body weight (stratum 1 contained the four lightest rats, and stratum 8 contained the four heaviest), and one rat from each stratum was assigned to each of the four HFD groups (HFD control and HFD+ NAR 50, 100, and 200 mg/kg). Similarly, we divided the NC rats (*n* = 16) into eight strata of two rats, and one rat from each stratum was assigned to each of the two NC groups (NC control and NC+ NAR 200 mg/kg). Each group therefore contained eight rats (*n* = 8/group) with a similar range of body weights. The sample size of 8 rats per group was selected for this exploratory study based on group sizes commonly used in previous experimental studies investigating the effects of NAR in metabolic models, which generally used 5–8 animals per group [31,95]. An 8-week HFD-induction period was selected based on previous rodent studies reporting HFD-associated metabolic and hepatic alterations within 8–12 weeks [48,96,97]. NAR was administered once daily by oral gavage between 9:00 and 10:00 a.m. for (4 weeks, weeks 9–12) at 5 mL/kg body weight (0.5 mL/100 g). We prepared working formulations each day at 10, 20, and 40 mg/mL to deliver 50, 100, and 200 mg/kg/day. The administered volume was recalculated weekly from each animal’s most recent body weight. The NC and HFD vehicle-control groups received an equal volume of the vehicle alone (90% physiological saline, 5% DMSO, and 5% Tween 80) by oral gavage once daily on the same schedule.

### 4.7. Morphometric and Nutritional Parameters

We weighed food daily, and intake was calculated as food provided minus food remaining. Spilled food was collected and subtracted from the recorded intake. For group-housed rats, cage-level food consumption was divided by the number of rats and reported as g/rat/day. We calculated energy intake (kcal/rat/day) by multiplying food intake by the energy density of each diet (3.85 kcal/g for D12450J and 5.24 kcal/g for D12492). Body weight (BW) was recorded weekly and body weight gain (BWG) was calculated as the difference between final and initial BW. At the end of the study, body mass index (BMI) was calculated as BW (g)/length^2^ (cm^2^). BMI values of 0.45–0.68 g/cm^2^ were considered normal while obesity was defined as BMI > 0.68 g/cm^2^ in accordance with Novelli et al. [98].

### 4.8. Blood and Tissue Collection

At the end of the experiment, all rats were fasted for 12 h overnight with free access to water. Animals were anesthetized by the administration of combination of ketamine (90 mg/kg) and xylazine (10 mg/kg) [48]. Blood samples (1–2 mL) were collected through direct cardiac puncture immediately after induction of anesthesia and distributed in clot activator-gel tubes for serum separation and EDTA tubes for plasma samples. Subsequently, in serum separator tubes were allowed to clot at room temperature for 30 min. All samples were centrifuged at 2500× *g* for 15 min at 4 °C, then transferred into labelled Eppendorf tubes, and were stored at −80 °C until biochemical analysis. After weighing, the liver was carefully excised on ice and washed with normal saline; a portion of each was directly fixed in 10% neutral buffered formalin for histological analysis, while the remaining tissues were snap-frozen in liquid nitrogen and stored at −80 °C for additional analysis.

### 4.9. Preparation of Tissue Homogenates

For tissue homogenate, 200 mg of liver were homogenized rapidly in ice-cold phosphate-buffered saline (PBS) (pH = 7.4) in a combination of 900 µL PBS and 100 µL protease inhibitor. The homogenized tissues were then centrifuged at 10,000× *g* for 15–20 min at 4 °C. The resulting supernatant was then carefully transferred to Eppendorf tubes and stored at −20 °C until further analysis.

### 4.10. Determination of Total Protein Content of the Homogenate Supernatant

The total protein content of the homogenate supernatant was evaluated using the Bradford assay (Bio-Rad^®^, Hercules, CA, USA) [99,100].

### 4.11. Determination of Plasma Glucose, Insulin and HOMA-IR

Fasting plasma concentration of glucose was evaluated using glucose MR kit following the glucose oxidase-peroxidase (GOD-POD) enzymatic colorimetric method according to the manufacturer’s protocol (REF-1129015, Linear Chemicals, S.L.U., Montgat, Spain) using a spectrophotometer (Ultrospec 2100 Pro, Amersham Pharmacia Biotech, Biochrom Ltd., Cambridge, UK). Fasting plasma insulin was measured from the same sample by a rat-specific sandwich ELISA, (Cat. No. ELK 10406; ELK Biotechnology Co., Ltd., Wuhan, China), according to the manufacturer’s instructions using an ELISA microplate reader (HumaReader HS, Human, Ref. No. 16670, Wiesbaden, Germany), and expressed in ng/mL. Glucose and insulin were measured from the same plasma sample. Although, we did not record the exact interval between induction and sampling, but the procedure was identical for all animals. We calculated HOMA-IR for each animal from its own paired values [101], using the rat-adapted equation [102]:

HOMA-IR = [fasting insulin (ng/mL) × fasting glucose (mmol/L)]/(22.5 × 0.0403)

Firstly, we converted glucose in mg/dL to mmol/L by multiplying by 0.0555 according to kit manual. The constant 0.0403 converts insulin from ng/mL to µU/mL (1 µU/mL = 0.0403 ng/mL), so insulin in ng/mL was entered directly. At the end, the individual values were then summarized per group. HOMA-IR is an indirect index of insulin resistance that has been found to agree moderately with the insulin tolerance test in high-fat diet-fed Wistar rats [103]. In the present study, we used it only as a relative estimate and not as a substitute for clamp measurements.

### 4.12. Measurements of Serum and Hepatic Lipid Profile

The serum and liver were measured using the enzymatic-colorimetric method assay kits for triglyceride (TG) (Cat. No. CE-BC- 261-M), total cholesterol (TC) (Cat. No. E-BC-K109-M), high-density lipoprotein cholesterol (HDL-C) (Cat. No. E-BC-K221-M), and low-density lipoprotein cholesterol (LDL-C) (Cat. No. E-BC-K205-M) according to the manufacturers’ protocol (Elabscience Biotechnology Co., Ltd., Wuhan, China).

### 4.13. Liver Function Tests

Concentrations of Alanine aminotransferase (ALT) (Cat. No. 007-100), Aspartate Aminotransferase (AST) (Cat. No. 015-100), and Gamma-glutamyl transferase GGT (Cat. No. 035-150) in serum were determined by a kinetic UV method according to the protocol of the kit manufacturer (United Diagnostics Industry (UDI), Dammam, Saudi Arabia) using a spectrophotometer (Ultrospec 2100 Pro, Amersham Pharmacia Biotech, Biochrom Ltd., Cambridge, UK).

### 4.14. Measurements of Oxidative Stress and Inflammation in Hepatic Homogenates

Antioxidant defense parameters were assessed by measuring reduced glutathione (GSH) levels (Cat. No. E-BC-K030-M), glutathione peroxidase (GSH-Px) (Cat. No. E-BC-K096-M), total superoxide dismutase (T-SOD) (Cat. No. E-BC-K019-M), and catalase (CAT) (Cat. No. E-BC-K031-M) by colorimetric assay kits from Elabscience Biotechnology Co., Ltd. (Wuhan, China). Rat tumor necrosis factor-α (TNF-α) (Cat. No. ELK1396), interleukin-6 (IL-6) (Cat. No. ELK1158), interleukin-10 (IL-10) (Cat. No. ELK1144), and interleukin-1β (IL-1β) (Cat. No. ELK1272) ELISA kits were purchased from ELK (Wuhan) Biotechnology Co., Ltd. (Wuhan, China).

### 4.15. Quantitative Gene Expression Analysis by (Real-Time PCR)

For the assessment of mRNA expression of hepatic peroxisome proliferator-activated receptor gamma (PPARγ), ChREBP, SREBP-1c, SIRT1, Nrf2, NF-κB, and β-actin, total RNA was extracted from the frozen (−80 °C) liver tissue using the RNAbler- Cells and Tissue Kit (Cat. No. RE95050, Haven Scientific, Jeddah, KSA) according to the manufacturer’s instructions. RNA concentrations and purity were assessed spectrophotometrically using a NanoDrop 2000 spectrophotometer (Thermo Fisher Scientific, Waltham, MA, USA) based on the A260/A280 ratio.

For reverse transcription, 1 μg of total RNA from each sample was converted to complementary DNA (cDNA) using the RT Ace First Strand cDNA Synthesis Kit (Cat. No. PCR3005, Haven Scientific, Jeddah, KSA) with random hexamer primers in a final reaction volume of 20 μL. Reverse transcription was performed using a GeneAmp PCR System 9700 (Thermo Fisher Scientific, Waltham, MA, USA). The synthesized cDNA was subsequently stored at −80 °C until quantitative real-time PCR analysis.

Quantitative real-time PCR was performed using the Applied Biosystems 7500 PCR System (7500 Fast; Applied Biosystems, Foster City, CA, USA). Each 20 μL PCR reaction contained 10 μL of EverGreen Universal qPCR Master Mix (Cat. No. PCR5501, Haven Scientific, Jeddah, KSA), 7 μL of PCR-grade water, 0.5 μL of each forward and reverse primer, and 2 μL of cDNA. Amplification was performed with an initial denaturation at 95 °C for 5 min, followed by 40 cycles of 95 °C for 15 s and 60 °C for 1 min. The expression of *Nfe2l2* (Nrf2), *Pparg* (PPARγ), *Srebf1* (SREBP-1c), *Sirt1* (SIRT1), *Mlxipl* (ChREBP), and *Rela* (NF-κB p65) was determined. The NF-κB primers specifically targeted the *Rela* gene encoding the NF-κB p65 subunit (RefSeq NM_199267.2). The SREBP-1c primers targeted *Srebf1* (RefSeq NM_001276707.1), whereas the ChREBP primers targeted *Mlxipl* (RefSeq NM_001393706.1). Primer sequences and accession numbers are provided in Table 7.

Primer specificity was assessed based on the corresponding rat RefSeq target sequences. Melting-curve analysis was performed following amplification to evaluate the specificity of the PCR products. No-template controls (NTCs) and no-reverse-transcription (−RT) controls were included to assess nonspecific amplification and potential genomic DNA contamination, respectively. Amplification efficiency was determined for each primer pair using standard-curve analysis. 

β-actin (*Actb*) was used as the endogenous reference gene, and its expression stability across the experimental groups was evaluated using the animal-level Ct values before normalization. Relative gene expression was calculated using the 2^^−ΔΔCt^ method [104] with β-actin as the endogenous reference gene and the designated control group as the calibrator. For each animal, ΔCt was calculated as ΔCt = Ct(_target gene_) − Ct(_β-actin_), and ΔΔCt was calculated as ΔΔCt = ΔCt(_sample_) − ΔCt(_calibrator_). Relative gene expression was expressed as 2^^−ΔΔCt^. Each sample was analyzed in technical triplicate, and the mean Ct value of the three technical replicates was used for subsequent calculations. Accordingly, statistical analyses were performed using animal-level ΔCt values, whereas 2^^−ΔΔCt^ values were used for presentation of relative gene expression.

### 4.16. Histopathological Assessment and Scoring

A small part of 10% neutral-buffered formalin-fixed liver specimens from each group were dehydrated in an alcohol series, cleared in xylene, and paraffin block-embedded. After routine processing, paraffin sections of each tissue were cut into 4 μm sections, placed on glass slides, and stained with Hematoxylin and eosin (H&E) stain. H&E staining was examined using an optical microscope (H550S; Nikon, Tokyo, Japan) under 400× magnification. Liver sections were assessed using a histopathological scoring system for liver damage [105]. Histological evaluation was performed for 8 animals in each group. For each animal, “3 liver sections were examined, and two microscopic fields per section were evaluated, yielding six fields per animal.” A pathologist blinded to group allocation scored each field for “macrovesicular steatosis, microvesicular steatosis, cytoplasmic degeneration, infiltrative, hemorrhage, and edema,” using a 4-point scale (0 = none, 1 = mild, 2 = moderate, 3 = severe). For each animal, the scores from all fields were averaged into a single value, and this animal-level average, rather than individual fields, was used for the statistical comparisons between groups. Liver tissue was obtained from the median lobe of each rat.

### 4.17. Statistical Analysis

All data were analyzed using the GraphPad Software, version 10.6.1 (San Diego, CA, USA). All the results were expressed as mean ± standard deviation (SD). Normality was assessed by the Shapiro–Wilk test. Data were analyzed using one-way analysis of variance (ANOVA), and group differences were assessed by Tukey’s post-hoc test. Histopathological scoring data, which were ordinal, were analyzed using the Kruskal–Wallis test, followed by Dunn’s multiple-comparisons test. Differences were considered significant at *p* < 0.05. No animals died. We analyzed body weight, food intake, and histopathology in all animals for 8 rats per group. Biochemical analyses used six animals per group, because the homogenate volume was insufficient for some animals. To keep group sizes equal, two animals per group were excluded from all biochemical measurements. Exclusion was based on sample availability, not on any measured value. No values were imputed and no outliers were removed.

## 5. Conclusions

The present study demonstrates the hepatoprotective effect of NAR against the metabolic disorders and liver damage induced by the HFD. We observed that NAR treatment was associated with improvement in hepatic lipid accumulation and several metabolic parameters, accompanied by lower expressions of ChREBP, SREBP-1c, and PPARγ, and higher SIRT1 expression. Our findings also demonstrated its association with improvements in antioxidant and inflammatory markers accompanied by higher Nrf2 and lower NF-κB mRNA expression. Thus, NAR presents promising evidence for its potential application in metabolic liver diseases. Further studies are needed to validate its therapeutic value and to address the molecular mechanisms responsible for its valuable effects.

## Figures and Tables

**Table 1:** Body weight, BMI, food and calorie intake, and liver weight and index across experimental groups.

	NC	NC+ 200 mg/kg NAR	HFD	HFD+ 50 mg/kg NAR	HFD+ 100 mg/kg NAR	HFD+ 200 mg/kg NAR
Initial BW (g) (Week 0)	152.8 ± 11.2	151.8 ± 12.4	154.1 ± 13.4	151.6 ± 9.0	152.9 ± 13.2	152.6 ± 11.0
Body Weight (g) (Week 8)	299.5 ± 4.7	295.3 ± 30.3	328.9 ± 16.8	313.4 ± 28.4	315.7 ± 40.3	317.6 ± 35.6
Final BW (g) (Week 12)	346.3 ± 41.8	342.6 ± 27.1	474.5 ± 46.7 ^ab^	441.4 ± 52.3 ^ab^	416.0 ± 39.1 ^abc^	377.8 ± 16.5 ^cd^
Weight change (from week 9–12)	28.9 ± 45.9	31.6 ± 44.3	138.9 ± 60.8 ^ab^	118.9 ± 56.2 ^ab^	89.0 ± 19.8	50.5 ± 47.8 ^c^
BMI (g/cm^2^)	0.52 ± 0.07	0.54 ± 0.07	0.73 ± 0.08 ^ab^	0.66 ± 0.09 ^ab^	0.62 ± 0.04 ^ac^	0.59 ± 0.02 ^c^
Avg. daily food intake (g/rat/day)	22.0 ± 1.80	18.9 ± 1.80 ^a^	20.2 ± 1.12	19.1 ± 0.49 ^a^	18.7 ± 0.92 ^a^	18.5 ± 0.80 ^ac^
Calorie intake (kcal/rat/day)	84.8 ± 6.91	72.7 ± 6.93 ^a^	105.8 ± 5.87 ^ab^	100.1 ± 2.54 ^ab^	98.2 ± 4.81 ^ab^	96.9 ± 4.22 ^abc^
Liver weight (g)	8.80 ± 0.87	9.47 ± 1.22	16.6 ± 1.46 ^ab^	12.9 ± 1.10 ^abc^	10.9 ± 1.53 ^acd^	9.55 ± 1.0 ^cd^
Liver index (%)	2.55 ± 0.17	2.76 ± 0.26	3.51 ± 0.30 ^ab^	2.94 ± 0.15 ^ac^	2.61 ± 0.12 ^cd^	2.53 ± 0.21 ^cd^

Values in the table represent the mean ± SD. Significance level was presented as *p* < 0.05. Statistical comparisons are denoted by letters as follows: (a) compared to control, (b) compared to control treated with NAR (200 mg/kg), (c) compared to HFD, (d) compared to HFD treated with NAR (50 mg/kg). A letter on a bar means that group differs significantly (*p* < 0.05) from the group listed for that letter. Abbreviations: BW: body weight; NC: normal control; HFD: high-fat diet; NAR: naringenin.

**Table 2:** Alterations in serum liver enzymes, fasting plasma glucose, insulin and HOMA-IR of rats in all groups.

	NC	NC+ 200 mg/kg NAR	HFD	HFD+ 50 mg/kg NAR	HFD+ 100 mg/kg NAR	HFD+ 200 mg/kg NAR
ALT (U/L)	34.9 ± 2.9	31.0 ± 3.3	51.3 ± 4.3 ^ab^	46.7 ± 4.9 ^ab^	41.4 ± 7.3 ^bc^	39.6 ± 2.1 ^bc^
AST (U/L)	24.9 ± 4.4	20.7 ± 4.8	56.1 ± 5.6 ^ab^	49.1 ± 3.3 ^ab^	39.2 ± 3.3 ^abcd^	32.0 ± 3.9 ^bcd^
GGT (U/L)	36.1 ± 4.5	33.8 ± 2.7	66.3 ± 5.0 ^ab^	58.6 ± 4.5 ^abc^	50.9 ± 3.8 ^abcd^	42.2 ± 3.7 ^bcde^
Fasting glucose (mmol/L)	6.19 ± 0.60	5.43 ± 0.43	8.58 ± 0.70 ^ab^	8.19 ± 0.64 ^ab^	7.22 ± 0.37 ^abcd^	6.74 ± 0.39 ^bcd^
Fasting insulin (ng/mL)	1.10 ± 0.13	0.96 ± 0.28	2.49 ± 0.33 ^ab^	2.20 ± 0.33 ^ab^	1.82 ± 0.22 ^abc^	1.40 ± 0.31 ^cd^
HOMA-IR	7.4 ± 1.4	6.1 ± 1.7	24.4 ± 4.1 ^ab^	20.4 ± 3.6 ^ab^	14.0 ± 1.3 ^abcd^	10.5 ± 2.5 ^cd^

Values in the table represent the mean ± SD. Significance level was presented as *p* < 0.05. Statistical comparisons are denoted by letters as follows: (a) compared to control, (b) compared to control treated with NAR (200 mg/kg), (c) compared to HFD, (d) compared to HFD treated with NAR (50 mg/kg), and (e) compared to HFD treated with NAR (100 mg/kg). A letter on a bar means that group differs significantly (*p* < 0.05) from the group listed for that letter. Abbreviations: NC: normal control; HFD: high-fat diet; NAR: naringenin; ALT: alanine transaminase; AST: aspartate transaminase; GGT: gamma-glutamyl transferase; HOMA-IR: homeostasis model assessment of insulin resistance.

**Table 3:** Changes in serum and hepatic lipid profiles of rats in all groups.

	NC	NC+ 200 mg/kg NAR	HFD	HFD+ 50 mg/kg NAR	HFD+ 100 mg/kg NAR	HFD+ 200 mg/kg NAR
Serum lipid profile						
TG (mmol/L)	1.5 ± 0.12	1.4 ± 0.10	2.7 ± 0.30 ^ab^	2.6 ± 0.28 ^ab^	2.2 ± 0.31 ^abc^	1.7 ± 0.32 ^cde^
TC (mmol/L)	1.7 ± 0.35	1.9 ± 0.32	3.7 ± 0.41 ^ab^	3.1 ± 0.48 ^ab^	2.9 ± 0.25 ^bc^	2.2 ± 0.38 ^cde^
LDL-c (mmol/L)	3.1 ± 0.53	2.9 ± 0.45	4.0 ± 0.51 ^ab^	3.9 ± 0.21 ^ab^	3.3 ± 0.54 ^c^	3.0 ± 0.35 ^cd^
HDL-c (mmol/L)	1.14 ± 0.13	1.16 ± 0.11	0.95 ± 0.06 ^ab^	1.06 ± 0.06	1.06 ± 0.14 ^c^	1.14 ± 0.11 ^c^
Liver lipid profile						
TG (µmol/g protein)	2.0 ± 0.29	1.9 ± 0.17	3.7 ± 0.40 ^ab^	3.5 ± 0.38 ^ab^	2.9 ± 0.19 ^abc^	2.4 ± 0.50 ^cd^
TC (mmol/g protein)	6.4 ± 0.84	5.6 ± 0.64	9.5 ± 1.1 ^ab^	8.4 ± 0.64 ^ab^	7.3 ± 0.61 ^bc^	6.8 ± 0.84 ^cd^
LDL-c (mmol/g protein)	6.2 ± 0.61	6.0 ± 0.34	9.9 ± 0.70 ^ab^	8.9 ± 0.88 ^ab^	7.6 ± 0.80 ^bc^	6.9 ± 1.6 ^cd^
HDL-c (mmol/g protein)	3.0 ± 0.18	3.1 ± 0.29	2.2 ± 0.38 ^ab^	2.5 ± 0.23 ^b^	2.7 ± 0.30 ^c^	2.9 ± 0.39 ^c^

Values in the table represent the mean ± SD. Significance level was presented as *p* < 0.05. Statistical comparisons are denoted by letters as follows: (a) compared to control group, (b) compared to control group treated with NAR (200 mg/kg), (c) compared to HFD group, (d) compared to HFD rats treated with NAR (50 mg/kg), and (e) compared to HFD rats treated with NAR (100 mg/kg). A letter on a bar means that group differs significantly (*p* < 0.05) from the group listed for that letter. Abbreviations: NC: normal control; HFD: high-fat diet; NAR: naringenin; TG: triglyceride; TC: total cholesterol; LDL-c: low-density lipoprotein cholesterol; HDL: high-density lipoprotein cholesterol.

**Table 4:** Effects of NAR on histopathological score of liver damage.

Groups	Macro Vesicular Steatosis	Micro Vesicular Steatosis	Cytoplasmic Degeneration	Infiltrative	Hemorrhage	Edema	Total
NC	0	0	0	0	0	0	0
NAR	0	0	0	0	0	0	0
HFD	2.6 ± 0.5 ****^a^	2.7 ± 0.4 ****^a^	2.6 ± 0.5 ****^a^	2.7 ± 0.4 ****^a^	2.3 ± 0.5****^a^	1.7 ± 0.4 ***^a^	14.6 ± 0.2 ****^a^
HFD+NAR 50 mg/kg	2.4 ± 0.5 ***^a,^***^b^	2.3 ± 0.7 **^a,^**^b^	2.2 ± 0.6 ***^a,^***^b^	2.3 ± 0.7 **^a,^**^b^	1.6 ± 0.5 **^a,^**^b^	1.7 ± 0.6 ***^a,^***^b^	12 ± 0.8 ***^a^
HFD+NAR 100 mg/kg	2 ± 0.5 *^a,^*^b^	2.2 ± 0.6 **^a,^**^b^	1.8 ± 0.6 *^a,^*^b^	2 ± 0.7 *^a,^*^b^	1.5 ± 0.5 **^a,^**^b^	1.6 ± 0.5 **^a,^**^b^	10.5 ± 0.7 *^a^
HFD+NAR 200 mg/kg	2 ± 0.8 *^a,^*^b^	1.8 ± 0.9 *^a,^*^b^	1.6 ± 0.7 *^a,^*^b^	1.7 ± 0.8 *^a,^*^b^	1.3 ± 0.5 *^a,^*^b^	1.3 ± 0.5 *^a,^*^b^	9.2 ± 0.5 **^b^

Data is presented as mean ± SD; Kruskal–Wallis test followed by Dunn’s test; *p* < 0.05 considered significant; superscript a: significant vs. control, superscript b: significant vs. HFD, * *p* < 0.05, ** *p* < 0.01, *** *p* < 0.001, **** *p* < 0.0001. Abbreviations: NC: normal control; HFD: high-fat diet; NAR: naringenin.

**Table 5:** Formula of the standard diet (SD) and high-fat diet (HFD).

	SD (D12450J) [92]		HFD (D12492) [93]	
	gm%	kcal%	gm%	kcal%
Protein	19.2	20	26	20
Carbohydrate	67.3	70	26	20
Fat	4.3	10	35	60
Total		100		100
kcal/g	3.85		5.24	
Ingredient	Gm	Kcal	gm	Kcal
Casein, 30 Mesh	200	800	200	800
L-Cystine	3	12	3	12
Corn Starch	506.2	2024.8	0	0
Maltodextrin 10	125	500	125	500
Sucrose	68.8	275.2	68.8	275
Cellulose, BW200	50	0	50	0
Soybean Oil	25	225	25	225
Lard *	20	180	245	2205
Mineral Mix S10026	10	0	10	0
DiCalcium Phosphate	13	0	13	0
Calcium Carbonate	5.5	0	5.5	0
Potassium Citrate, 1 H_2_O	16.5	0	16.5	0
Vitamin Mix V10001	10	40	10	40
Choline Bitartrate	2	0	2	0
FD&C Yellow Dye #5	0.04	0	0	0
FD&C Red Dye #40	0	0	0	0
FD&C Blue Dye #1	0.01	0	0.05	0
Total	1055.05	4057	773.85	4057

* Typical analysis of cholesterol in lard = 72 mg/100 g. Cholesterol (mg)/4057 kcal = 216.4 cholesterol (mg)/kg = 279.6. #The U.S. Food and Drug Administration (FDA) certified color index number. The composition of both diets obtained online from the supplier’s webpage: Research Diets, Inc. (New Brunswick, NJ, USA).

**Table 6:** Experimental protocol of animal grouping, diets, and treatments.

Groups	Diets	Treatments
NC	Standard diet	Vehicle, p.o. last 4 weeks
NC+ 200 mg/kg NAR	Standard diet	NAR (200 mg/kg/d), p.o. last 4 weeks
HFD	High-fat diet	Vehicle, p.o. last 4 weeks
HFD+ 50 mg/kg NAR	High-fat diet	NAR (50 mg/kg/d), p.o. last 4 weeks
HFD+ 100 mg/kg NAR	High-fat diet	NAR (100 mg/kg/d), p.o. last 4 weeks
HFD+ 200 mg/kg NAR	High-fat diet	NAR (200 mg/kg/d), p.o. last 4 weeks

Abbreviations: NC: normal control; HFD: high-fat diet; NAR: naringenin.

**Table 7:** List of the primer sequences for quantitative real-time PCR analysis.

Gene	Forward (5′-3′)	Reverse (5′-3′)	Accession Number
Nrf2	AAAATCATTAACCTCCCTGTTGAT	CGGCGACTTTATTCTTACCTCTC	NM_031789
PPAR-γ	CCAGTTTCGATCCGTGGAAG	CTTTTGAGGAACTCCCTGGTCA	NM_001145366.1
SREBP-1c	GAGGCCACAGTGCTCATTCT	CTGGCCTCACTCATTGTCGT	NM_001276707.1
NF-κB	ACGACACCTCTACACATAGCA	CCTCATCTTCTCCAGCCTTCT	NM_199267.2
SIRT1	AGGGAACCTCTGCCTCATCT	AAGCTGCCTAGCTGCTTGAA	NM_001372090.1
ChREBP	GAAGACCCAAAGACCAAGATGC	TCTGACAACAAAGCAGGAGGTG	NM_001393706.1
β-actin	CCACCATGTACCCAGGCATT	CCTAGAAGCATTTGCGGTGC	NM_031144.3

## Data Availability

The datasets used and analysed during the current study are available from the corresponding author upon reasonable request.
